# Case Report: presence of granulosa cells in the uterine tissue of a spayed Labrador Retriever bitch

**DOI:** 10.3389/fvets.2026.1788539

**Published:** 2026-03-27

**Authors:** Maria Pereira, Marta Giacomazzo, Lluis Ferré-Dolcet, Stefano Romagnoli, Ranieri Verin

**Affiliations:** 1 Department of Animal Medicine, Production and Health (MAPS), University of Padova, Legnaro, Italy; 2 Department of Comparative Biomedicine and Food Science (BCA), University of Padova, Legnaro, Italy; 3 Private Practitioner, Barcelona, Spain; 4 Department of Veterinary Sciences, University of Pisa, Pisa, Italy

**Keywords:** embryological defect, female dog, granulosa cells, ovary remnant, post-sterilization estrus

## Abstract

A 10-year-old Labrador Retriever bitch, spayed at 2 years of age, was presented to our veterinary care facility with a history of vulvar swelling, vaginal discharge, and male attraction for over 1 month. Vaginoscopy revealed edema and reddening of the vaginal mucosa. Vaginal cytology was consistent with estrus, while serum progesterone levels remained at baseline. On abdominal ultrasound, the uterus was hyperplastic, and fluid-filled cysts were visualized at both ovarian anatomic sites. Hysterectomy and excision of tissue from both ovarian sites were performed 10 days after the initial presentation. The gross appearance of the uterus was consistent with cystic endometrial hyperplasia (CEH), confirmed by histopathology. Ovaries were not detected microscopically; however, small multifocal nests, occasionally forming pseudocysts or follicle-like structures, were observed in continuity with the uterine horns and embedded within the myometrium. These nests consisted of well-differentiated round to polygonal and occasionally spindle-shaped cells that underwent luteinization. These cells were immunopositive for inhibin-*α*, weakly immunopositive for progesterone receptor, and immunonegative for estrogen receptor α. Serum anti-Müllerian hormone (AMH) was elevated before hysterectomy and returned to basal levels afterward. Based on these findings, the cells were identified as granulosa cells embedded within the uterine tissue. We propose an embryological defect or iatrogenic seeding of ovarian tissue within the uterus as the most likely differential diagnosis, although neoplastic granulosa cells could not be completely ruled out, even though this is extremely unlikely. All clinical signs resolved after surgery, and the bitch did not exhibit any recurrence of estrus behavior until her death, approximately 2.5 years later.

## 1 Introduction

Signs of estrus in a sterilized bitch are highly indicative of ovarian remnant syndrome, resulting from the presence of functional residual ovarian tissue after ovariectomy or ovariohysterectomy (1). The time gap between gonadectomy and the first manifestation of estrus signs is variable and can range from a few months to several years (1, 2). In the bitch, the diagnosis is made through vaginal cytology and serum progesterone assay. If the animal is examined while exhibiting heat behavior, vaginal cytology should be characterized by progressive (proestrus) or complete (estrus) epithelial cell keratinization. When complete keratinization is achieved, a serum progesterone level of >2.0 ng/mL will confirm luteal activity (1). If a diagnosis is believed when the bitch is not in heat, other means, such as luteinizing hormone (LH) or anti-Müllerian hormone (AMH) assays, can be used (3). Surgically excised ovarian remnant tissue should always be assessed histologically to confirm the diagnosis and exclude other differentials causing estrus signs, such as a sex cord-stromal neoplasia (granulosa cell tumor) in ovarian remnants (4, 5). This neoplasia may be hormonally functional and associated with the secretion of estrogens and/or progesterone and/or inhibin (6–8, 33). Excision of the neoplastic tissue is typically curative (9). Vulvar discharge and vulvar swelling may also be caused by exogenous exposure to estrogen-containing compounds (e.g., creams and sprays) usually belonging to the owners (10, 11). This possibility should always be excluded by careful collection of the patient’s history.

The present case report describes the clinical development and resolution of a spayed bitch with vulvar swelling, vaginal discharge, and male attraction. The dog was initially diagnosed with vaginitis and later suspected of having ovarian remnant syndrome. However, laboratory, histopathological, and immunohistochemical findings revealed the presence of granulosa cells embedded within the uterine wall—independent of any ovarian tissue—which were identified as the most plausible source of the clinical signs.

## 2 Case description

Daisy, a 10-year-old spayed Labrador Retriever guide-dog bitch weighing 30 kg, was presented to the Veterinary Teaching Hospital in early February 2023 due to vulvar discharge and vulvar edema lasting over 1 month (day 0). The bitch had been surgically ovariectomized at 2 years of age. During the month prior to admission, Daisy began intensely licking her swollen vulvar region, and male dogs were increasingly attracted to her. The referring veterinarian diagnosed vaginitis based on vaginal cytology and normal urinalysis and treated it with a 2-week oral course of spiramycin and metronidazole together with probiotics. The clinical signs worsened, with the bitch exhibiting scant yellowish-to-reddish vulvar discharge during the days prior to referral. Clinical and blood examinations were unremarkable, and vaginal palpation did not reveal the presence of masses or strictures. Colposcopy with a vaginal speculum showed edematous and erythematous vaginal mucosa. Vaginal cytology was consistent with estrus, exhibiting more than 90% of keratinized epithelial cells, mostly nucleated, with few erythrocytes and no polymorphonuclear cells (Supplementary Figure 1).

**Figure 1:**
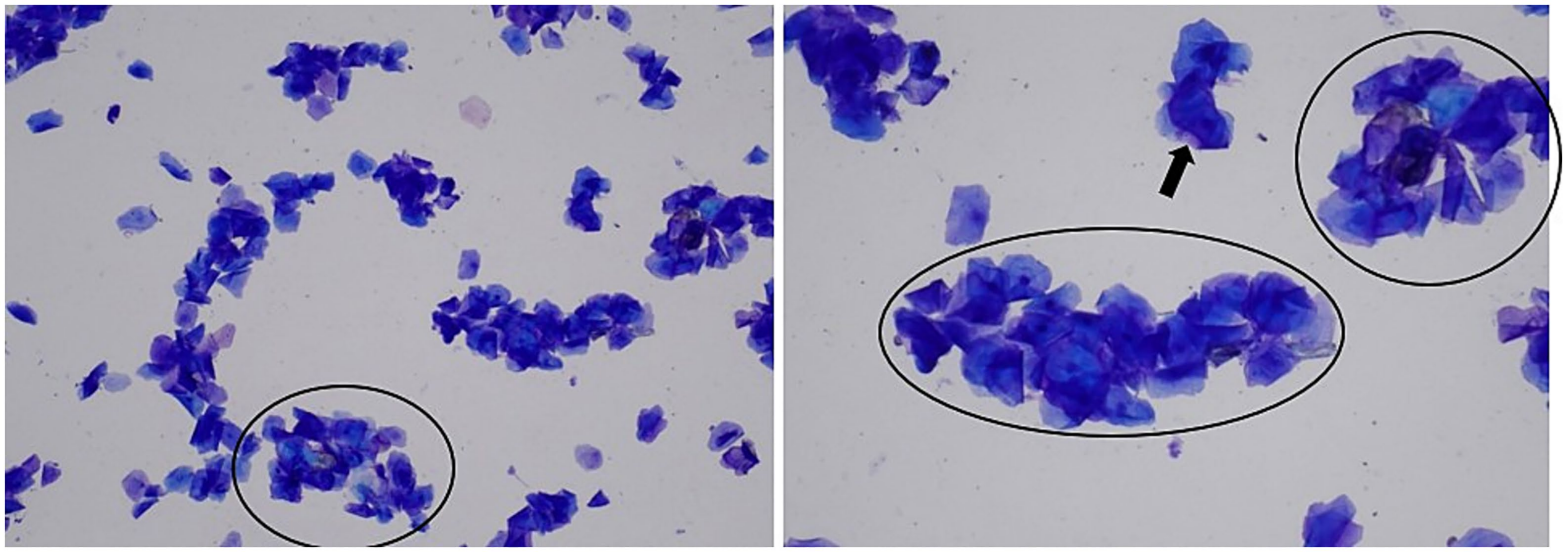
Daisy’s vaginal cytology 1 week post-presentation (day 7) indicative of advanced estrus. High cellularity, 100% of keratinization of epithelial cells, mostly without nucleus (black arrow), absence of erythrocytes, clearer background, and presence of small clusters of keratinized cells (black circles). Left—100x magnification; right—200x magnification.

After the anamnesis ruled out exposure to estrogen-containing products, the primary differential diagnoses included the presence of an ovarian remnant, either causing follicular growth, or generating a granulosa cell tumor, or an estrogen-secreting teratoma (12). Serum progesterone concentration was 0.26 ng/mL (AIA 360^TM^, Tosoh Bioscience, Japan). In bitches, a concentration below 1.0 ng/mL is considered basal since the LH peak, which precedes ovulation, is estimated to occur at progesterone values of 1.5–2.0 ng/mL (13). A week later (day 7), serum progesterone had increased to 0.51 ng/mL, and vaginal cytology showed a more advanced estrus stage (Figure 1).

**Figure 2:**
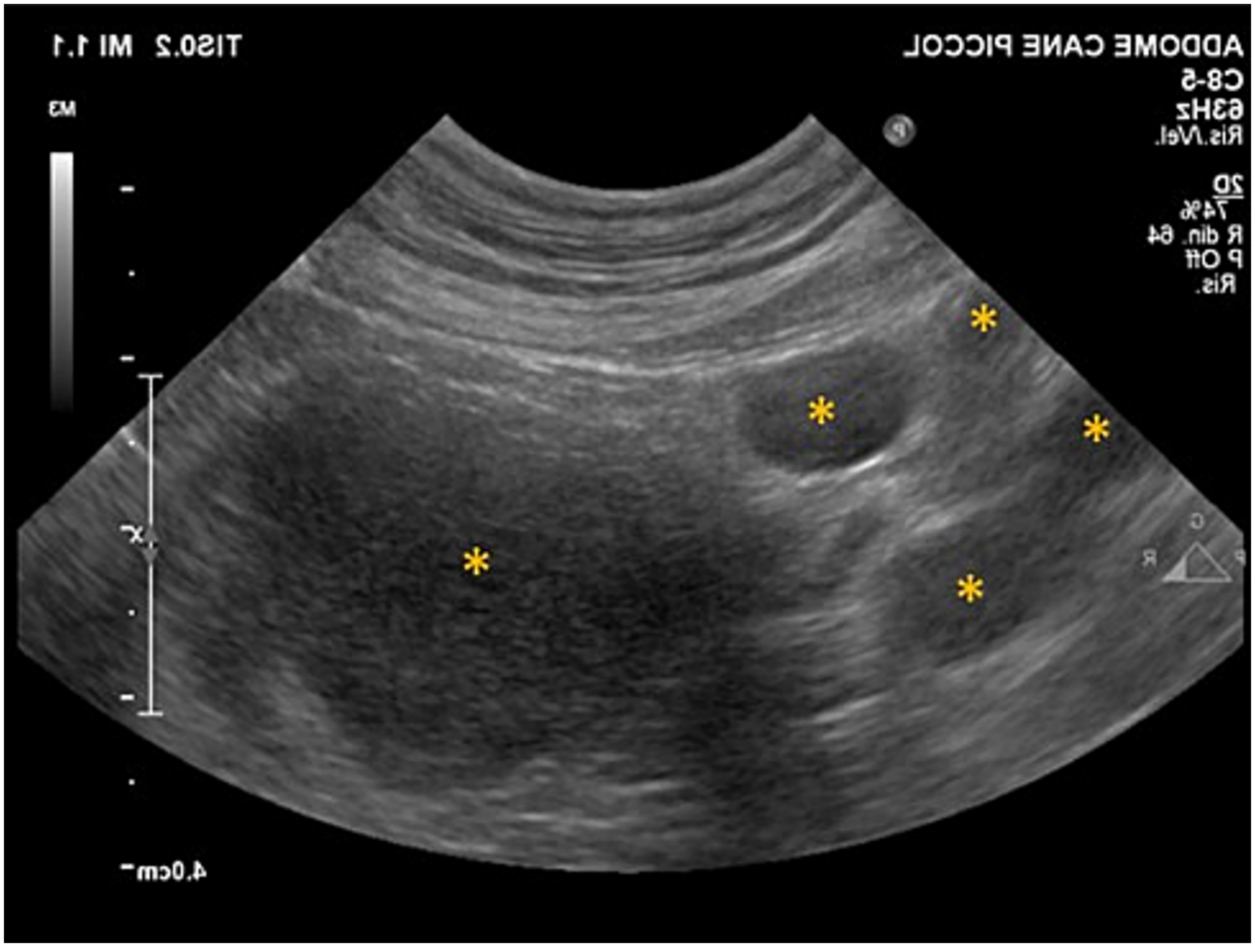
Ultrasonographic features of Daisy’s reproductive tract. Fluid-filled structures (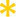) at the left ovarian anatomical site.

Hysterosonography was performed to detect the presence of ovaries or an ovarian remnant. The uterus exhibited an atypical appearance for a spayed bitch, with uterine diameter measuring 20.2 mm at the level of the horns. Several fluid-filled structures (Figure 2) were observed in both the left and right ovarian anatomical regions, with maximum diameters of 26.6 and 5.3 mm, respectively. The fluid-filled structures were initially presumed to represent multiple large ovarian cysts. Notwithstanding, the ultrasound images were also compatible with uterine dilations containing luminal fluid.

On day 10, an exploratory laparotomy was performed under general anesthesia. The bitch was pre-medicated with dexmedetomidine at a dosage of 3.0 μg/kg and methadone at a dosage of 0.2 mg/kg IM, and anesthesia was induced with propofol at a dosage of 3.0 mg/kg IV and maintained with isoflurane in 100% oxygen. The uterus had a gross appearance compatible with cystic endometrial hyperplasia (CEH). On the proximal poles of the uterine horns (the presumptive anatomical sites of the ovaries), the tissue was characterized by a white, edematous, and homogeneous appearance, displaying multifocal small cystic-like structures (Supplementary Figure 2). The whole reproductive tract was removed and sent for histopathological examination. Meloxicam at a dosage of 0.2 mg/kg IV was administered immediately after surgery. The patient was discharged later the same day, with meloxicam at a dosage of 0.1 mg/kg PO SID prescribed for the following 4 days. Serum progesterone, assayed immediately before surgery (day 10 from referral), was 0.29 ng/mL.

**Figure 3:**
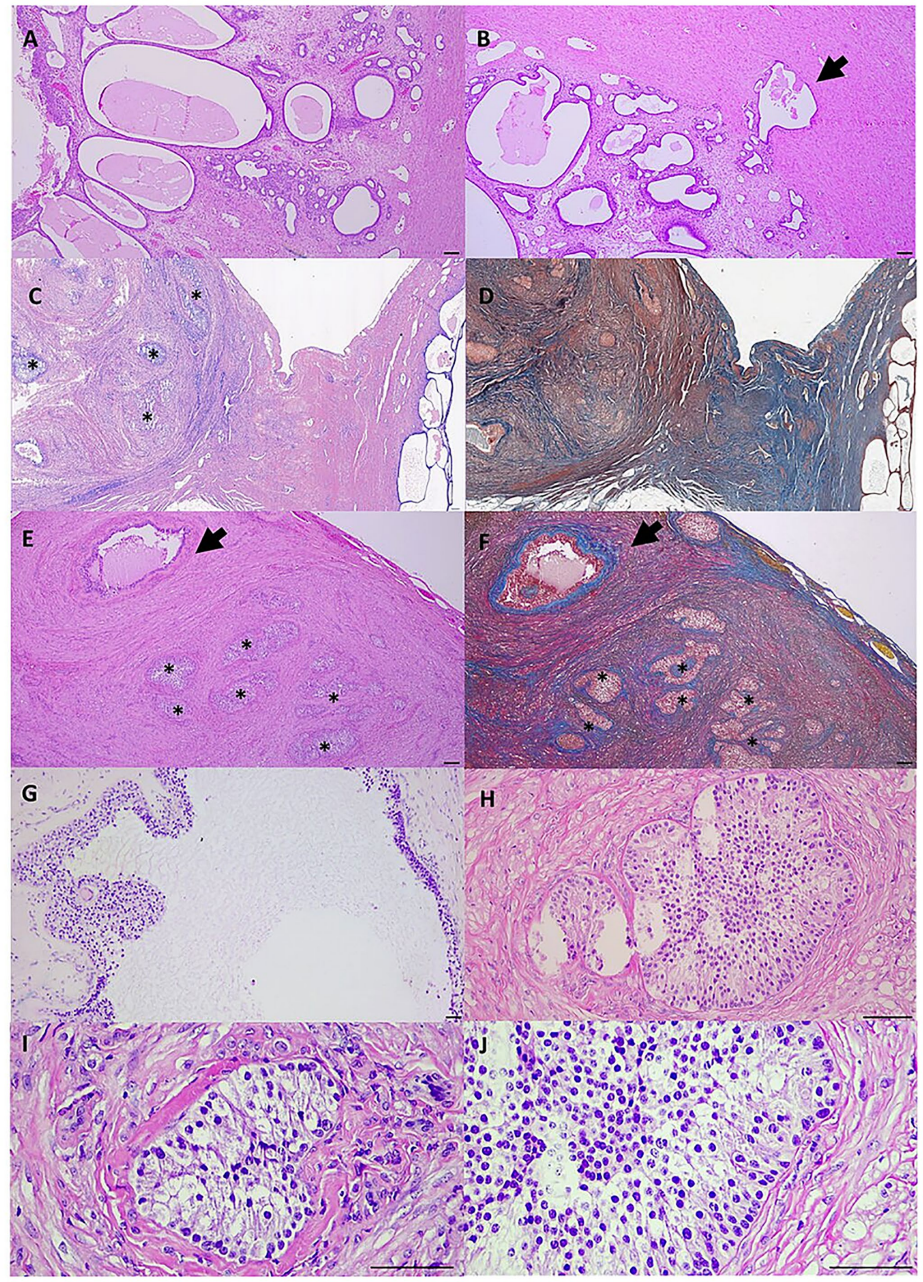
Canine uterine tissue, scale bar: 100 μm. **(A)** The uterine mucosa exhibits cystic endometrial hyperplasia. Objective (Ob): 4x (hematoxylin and eosin, H&E). **(B)** Occasional endometrial glands are detected transmurally, embedded within the myometrium (adenomyosis, arrow). Ob: 4x (H&E). **(C)** Close to the suspected anatomical site of the ovary, in contiguity with the cranial portion of the uterine horns, islands and nests of clear palisading cells resembling granulosa cells (asterisks) are embedded within fibrovascular stroma and smooth muscle cells. Ob: 2x (H&E). **(D)** The fibrovascular stroma/serosa and smooth muscle cells are distinctly visualized using Masson’s trichrome stain in blue and red, respectively. Ob: 2x, Masson’s trichrome. **(E,F)** At higher magnification, scattered embedded islands and nests of cells resembling granulosa cells (asterisks) can be seen, occasionally forming pseudocyst-like or follicle-like structures (possibly atretic follicles, arrows). Ob: 4x (H&E and Masson trichrome). **(G)** A frame of palisading cells resembling granulosa cells outlining a pseudocyst-like structure of bigger dimensions (possibly an atretic follicle). Ob: 10x (H&E). **(H)** The granulosa cell-like elements are palisading, polygonal, and occasionally spindle-shaped, with variably abundant, clear to glassy cytoplasm. Ob: 20x (H&E). **(I,J)** Higher magnification of granulosa cell-like elements. Ob: 40x (H&E).

The entire reproductive tract was fixed in 10% buffered formalin. Paraffin-embedded sections (4-μm thick) were stained with hematoxylin and eosin (H&E) and Masson’s trichrome and were evaluated by a board-certified pathologist (RV) and a veterinary pathologist (MG). Histologically, the uterine mucosa exhibited endometrial hyperplasia, characterized by hyperplastic, cystic, variably dilated, and occasionally interrupted endometrial glands, which were embedded in fibrous stroma (Figure 3A). Occasionally, endometrial glands were detected transmurally, embedded within the myometrium (adenomyosis, Figure 3B). No evidence of ovarian tissue was observed in numerous consecutive serial microcuts in the sections taken from the presumed anatomical sites of the ovaries. The examined tissue, extending from and continuous with the uterine horns (Figures 3C,D), was characterized by a fibrovascular stroma containing densely arranged smooth muscle cells, highlighted by Masson’s trichrome stain (Figures 3D,F) and interpreted as the myometrium. This layer was surrounded by the serosa, which stained blue with Masson’s trichrome. Within this stroma, scattered embedded islands or nests, along with cords of round to polygonal, occasionally spindle-shaped cells with variably abundant, clear-to-glassy cytoplasm, were observed (Figures 3H,I,J). These cells were occasionally peripherally palisading (Figures 3H,I,J) and morphologically resembled variably luteinized granulosa cells (Figures 3E,H), without evidence of surrounding ovarian follicles. Cellular atypia was absent or minimal, and no mitotic activity was observed. Occasional pseudocysts or follicle-like structures (possibly atretic follicles based on their morphology: the follicular wall was folded, and the follicular fluid space was collapsed rather than distended), of varying dimensions, filled with proteinaceous amorphous material, and lacking an epithelial or endothelial internal lining, were observed encased in the stroma and lined by palisading cells resembling degenerated granulosa cells (Figure 3G).

Immunohistochemistry (IHC) was performed on uterine tissue sections using a semi-automated immunostainer (Ventana BenchMark GX; Roche Diagnostics). Detailed information on the primary antibodies is provided in Table 1. The staining procedures were carried out according to the manufacturer’s instructions. Briefly, tissue sections were deparaffinized, and endogenous peroxidase activity was blocked using 3% hydrogen peroxide (UltraView Universal DAB Inhibitor; Roche Diagnostics). The sections were then incubated for 1 h with the appropriately diluted primary antibody in Ventana Antibody Diluent (Cat. No. 251–018/05261899001). After rinsing in a Tris-based reaction buffer (pH 7.6; Reaction Buffer, Roche Diagnostics), the sections were incubated with a secondary antibody cocktail consisting of goat anti-mouse IgG, goat anti-mouse IgM, and goat anti-rabbit IgG antibodies conjugated to a horseradish peroxidase (HRP)-conjugated polymer (UltraView Universal HRP Multimer; Roche Diagnostics). Immunoreactivity was visualized using 3,3′-diaminobenzidine (DAB) as the chromogen (UltraView Universal DAB Detection Kit; Roche Diagnostics). The sections were counterstained with hematoxylin. Negative controls were obtained by omission of the primary antibody, which was substituted with the antibody diluent.

**Table 1:** Specific information on the primary antibodies used for immunohistochemistry.

Antibody	Antibody clone	Species/Source	Dilution	Positive control
Anti-estrogen receptor alpha (ERα)	Clone 6F11 (Novocastra)	Mouse monoclonal	1:40	Normal canine uterus and uterus with CEH
Anti-progesterone receptor (PG)	Clone PR88 (BioGenex)	Mouse monoclonal	1:100	Normal canine uterus and uterus with CEH
Anti-inhibin-α (INHα)	Clone R1 (Serotec Co., Oxford, UK)	Mouse monoclonal	1:40	Normal canine ovary

CEH, cystic endometrial hyperplasia.

**Figure 4:**
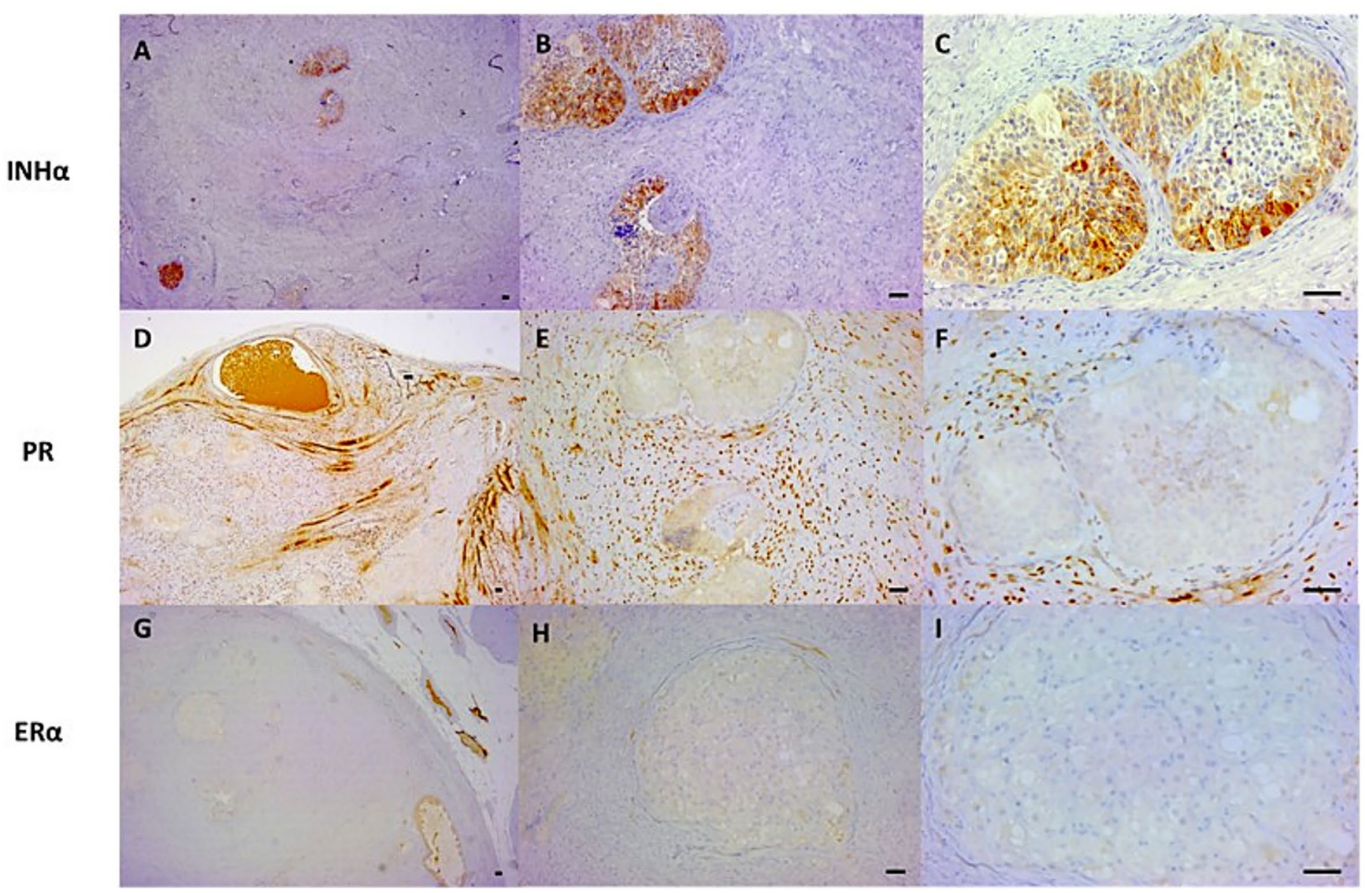
Immunohistochemical expression of INH*α*, PR, and ERα in the myometrium. The colorimetric reactions were developed using diaminobenzidine (DAB). **(A–C)** The islands of clear palisading cells show variable and moderate cytoplasmic immunostaining for INHα. Objective (Ob): 2.5X **(A)**, 10X **(B)**, and 20X **(C)**. **(D–F)** The islands of clear palisading cells are negative or focally very weakly positive for PR, whereas the smooth muscle cells and stromal cells in the myometrium show variable and moderate nuclear immunostaining. Ob: 2.5X **(D)**, 10X **(E)**, and 20X **(F)**. **(G–I)** The islands of clear palisading cells and the normal uterine tissues exhibit negative immunostaining for ERα. Ob: 2.5X **(G)**, 10X **(H)**, and 20X **(I)**. Scale bar = 80 μm.

The islands of putative granulosa cells exhibited diffuse cytoplasmic immunoreactivity for INHα, with only very weak and focally positive nuclear immunostaining for PR. Smooth muscle cells and stromal cells showed strong and specific PR immunolabeling. All tested tissues were immunonegative for ERα (Figure 4).

Based on histopathology results, the concentration of AMH (human AMH kit—validated for dogs, cats, and horses—IDEXX Milan, Italy) was measured in a sample obtained prior to surgery and stored frozen (5.79 pmol/L), confirming the presence of functional ovarian tissue. Concentrations of ≥ 0.50 pmol/L indicate the presence of functional (AMH-producing) ovarian tissue, according to the laboratory reference values.

Daisy had an uneventful postoperative recovery, with the disappearance of all clinical signs. The bitch was examined twice in the 7 months after surgery. During both follow-up moments, there was no vulvar discharge or edema, and the mucosa was light pink. Vaginal cytology was consistent with anestrus or sterilized status (Supplementary Figure 3). At the 7-month follow-up, blood was drawn for AMH measurement, yielding 0.30 pmol/L, a value compatible with a gonadectomized animal.

Afterward, follow-up was maintained through telephone contact with the owners until the death of the animal for unrelated causes, almost 2.5 years after the initial presentation.

## 3 Discussion

This study constitutes the first report of the presence of granulosa cells deeply embedded within the uterine tissue, capable of producing AMH and causing estrogenic-like alterations (vulvar swelling, vaginal discharge, male attraction, and keratinization of the vaginal epithelium).

Before obtaining the histological and immunohistochemical results, the primary differential diagnoses for this case were follicular cysts developed on bilateral ovarian remnants or a granulosa cell tumor arising from bilateral ovarian remnants. However, neither ovarian tissue nor ovarian remnants were identified, either macroscopically or histologically. At the level of the uterine horns’ extremities, nests and pseudocysts or follicle-like structures formed by clear palisaded granulosa-like cells were found embedded in smooth cells and fibrovascular stroma, as corroborated by Masson’s trichrome staining, in continuity with the uterine tissue, interpreted as the myometrium, and surrounded by the serosa. The pseudocysts or follicle-like structures (possibly atretic follicles) have partially contributed to the fluid-filled structures observed on ultrasound and macroscopically after hysterectomy.

Smooth muscle cells and stromal cells were immunopositive for PR, whereas the putative nests of granulosa cells expressed very weak and focal PR immunostaining. These findings are consistent with the observations of De Bosschere et al. (14) and Vermeirsch et al. (15), who found PR immunohistochemical expression in various histotypes of the normal canine uterus, including stromal cells and smooth muscle cells (14). These results further suggest that the smooth muscle cells observed originate from the uterus, while the weaker but immunopositive signal corresponds to the ovarian granulosa cell cords (15). Moreover, the PR staining intensity in ovarian follicles increases with follicle development (15). Based on these reports, PR immunolabeling was used to assess and confirm its expression, activity, and tissue origin. We can hypothesize that the putative granulosa cells underwent a process of luteinization, explaining the macroscopic and microscopic evidence of CEH and their arrangement in corpus luteum-like architecture. Nevertheless, the progesterone concentration in the peripheral blood was consistently basal despite minor fluctuations detected (from 0.26 to 0.51 to 0.29 ng/mL on days 0, 7, and 10, respectively), which are not clinically significant since they remain below 1.0 ng/mL (13). Minor fluctuations might be due to intra- and inter-assay variability (16).

Estrogen receptor alpha (ER*α*) was used, as it is commonly expressed in the reproductive tract (17). In our study, ERα immunoexpression was absent in all examined tissues. We hypothesize that the granulosa cells secreted estrogen, and the negative ERα immunostaining may reflect receptor downregulation due to sustained hormone exposure (18–20) or, similarly to patterns described in women (21), whose granulosa cells mainly express ERβ. Canine uterine cells might also predominantly express ERβ rather than ERα, which was the receptor targeted by the antibody used in this study.

The islands of cells found within the uterine tissue were not only morphologically consistent with granulosa cells but also specifically positive for INH-α, a reliable immunohistochemical marker of granulosa cells (22, 33), previously described in granulosa cell cords (34) and predominantly secreted during the luteal phase (23), thus confirming our suspicion. Theca cells were excluded morphologically and immunohistochemically, as they are INHα negative (22) and exhibit stronger PR staining (15), unlike the observed cells. Consequently, INHα was selected to confirm the presence of granulosa cells, since inhibin-positive immunostaining is a distinctive feature of both normal and neoplastic granulosa cells and reflects sex cord differentiation (24).

In summary, the use of these antibodies provided specific biological and diagnostic insights, confirming the identity of the granulosa cells (INHα), the presence of partial luteinization (PR), the reproductive origin of the tissue, and its hormonal expression (INHα and PR). Together, these markers supported our hypothesis and differential diagnosis, ruling out a non-sex cord origin for the island cells observed.

Granulosa cells are the only source of AMH in females (25, 26), and the elevated preoperative AMH, which returned to basal levels postoperatively, further supports that these cells found inside the uterus were granulosa cells.

This case shares some clinical and diagnostic features with reported canine ovarian remnant syndrome cases (2, 27), including vulvar swelling, discharge, male attraction, and the use of abdominal ultrasound and histopathology (2, 27). However, unlike those cases, no ovarian tissue or granulosa cell tumor was identified in the resected reproductive tract of our case.

The origin of the granulosa cells within the myometrium remains uncertain and may be due to: (a) an embryological defect (choristoma), (b) iatrogenic seeding during ovariectomy, or (c) neoplastic transformation, the latter being least likely.

An embryological developmental anomaly could result from the incomplete migration of primordial germ cells during early embryogenesis. The genital system derives from the intermediate mesoderm. Primordial germ cells migrate to the genital ridges of the embryo, medial to the embryological kidney, which consist of the early bipotential gonads. Females (XX embryos) lack the SRY gene. The SRY gene is located in the short arm of the Y chromosome and stimulates Sox-9 activity, determining the differentiation of stromal cells into Sertoli cells (28, 29). Conversely, in females, primordial germ cells evolve into granulosa cells to form the future ovary (30, 31). Concurrently, the mesonephric ducts regress, and the paramesonephric ducts continue to develop into the oviducts, uterine horns, body, and cranial vagina due to the absence of AMH (28, 31). Primordial germ cells may, however, follow inappropriate migratory pathways into extragonadal sites. These cells tend to degenerate, but persistence is possible (28). In our case, primitive germ cells could have been entrapped within the paramesonephric ducts and differentiated into granulosa cells embedded transmurally within the myometrium. Despite the likelihood of encountering an identical case being very low, similar mechanisms may underlie other pathological situations, highlighting the importance of reporting it.

Iatrogenic seeding was considered less likely, as superficial implantation would be expected; however, the granulosa cells in this case were located deeply within the myometrium, bilaterally, and surrounded only by the serosa. Neoplastic transformation, though improbable, would be compatible with deep, multifocal clusters, but the absence of atypia and metastatic features makes this unlikely. Extragonadal sex cord tumors have been described in male dogs and cats following castration, attributed to embryological ectopy or surgical transplantation (32). In our case, the absence of a primary ovarian tumor or a mass grossly evident at the time of the surgery, along with the histological features of the clusters of ectopic granulosa cells and the presence of only minimal atypia in this cellular population, renders the hypothesis of metastatic development extremely unlikely.

This case presented with several diagnostic challenges: ambiguous ultrasonographic findings suggestive of ovarian cysts, differentiation between ovarian remnant syndrome and ectopic ovarian tissue achieved with histology, absence of identifiable ovarian tissue histologically, basal progesterone despite CEH, and the extreme rarity of the condition. Although a definitive diagnosis was not achieved at surgery, a complete hysterectomy was curative. The decision was made intraoperatively after confirming the absence of macroscopic ovarian tissue, as isolated tissue excision could have necessitated a second surgery and limited histopathological evaluation. Owners had no interest in preserving reproductive function.

We recognize several limitations, particularly in the diagnostic approach. Estrogen concentration was not assayed during the period of estrus signs and keratinization of vaginal epithelium, and computed tomography was not performed prior to surgery, which might have confirmed the absence of independent ovarian tissue. Additionally, immunohistochemical evaluation was qualitative, with no quantitative assessment (e.g., H-score or digital image analysis), potentially limiting the precision of receptor expression estimation. A further limitation is the availability of validated antibodies for the detection of canine granulosa cells.

## 4 Conclusion

This clinical case reports the presence of granulosa cells within the uterus of a bitch, an occurrence that represents an extremely rare condition. However, no doubts remain regarding the nature of the granulosa cells, which were established through determination of the AMH serum concentration and specific INH-*α* tissue immunolabeling. The same cannot be said for the pathogenetic mechanisms underlying this phenomenon. Three proposed scenarios are discussed here, but, at this stage, it is not possible to draw a solid conclusion on which one is the most likely. Nevertheless, this condition was fully resolved through hysterectomy, and the bitch remained healthy and did not show recurrence of heat in the 2.5 years following surgery.

## Data Availability

The original contributions presented in the study are included in the article/Supplementary material, further inquiries can be directed to the corresponding author.
