# Supplementary material for: Case Report: presence of granulosa cells in the uterine tissue of a spayed Labrador Retriever bitch
Source: Front Vet Sci. 2026 Mar 27;13:1788539. doi: 10.3389/fvets.2026.1788539 (PMC13065685; doi:10.3389/fvets.2026.1788539)
Supplement: Supplementary file 1 [file Data_Sheet_1.DOCX]

Supplementary Material

# Supplementary Figures and Tables

## Supplementary Figures


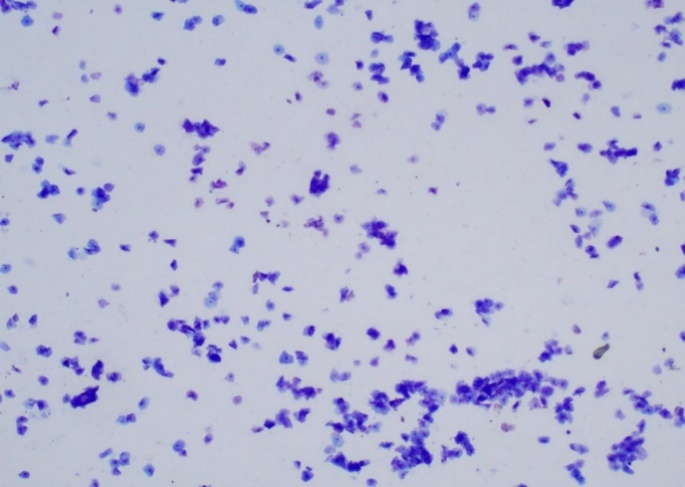


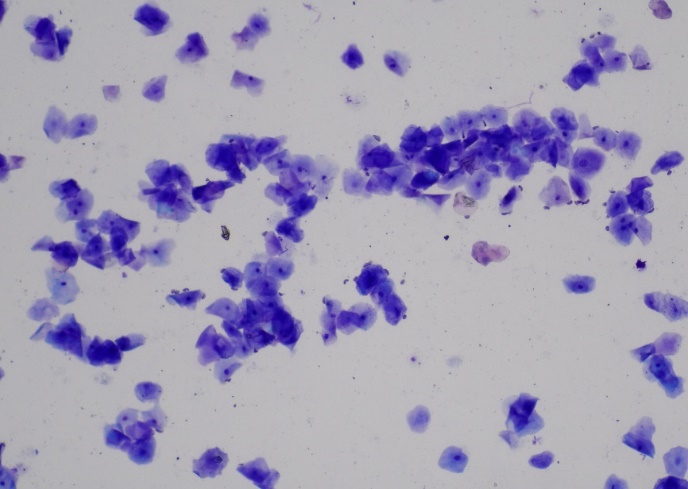


**Supplementary Figure 1.** Daisy’s vaginal cytology at presentation (Day 0). High cellularity, over 90% of keratinization of epithelial cells, mostly with nucleus (black arrow), sparse erythrocytes and absence of polymorphonuclear cells, compatible with estrus. Left - 40x magnification; Right – 100x magnification


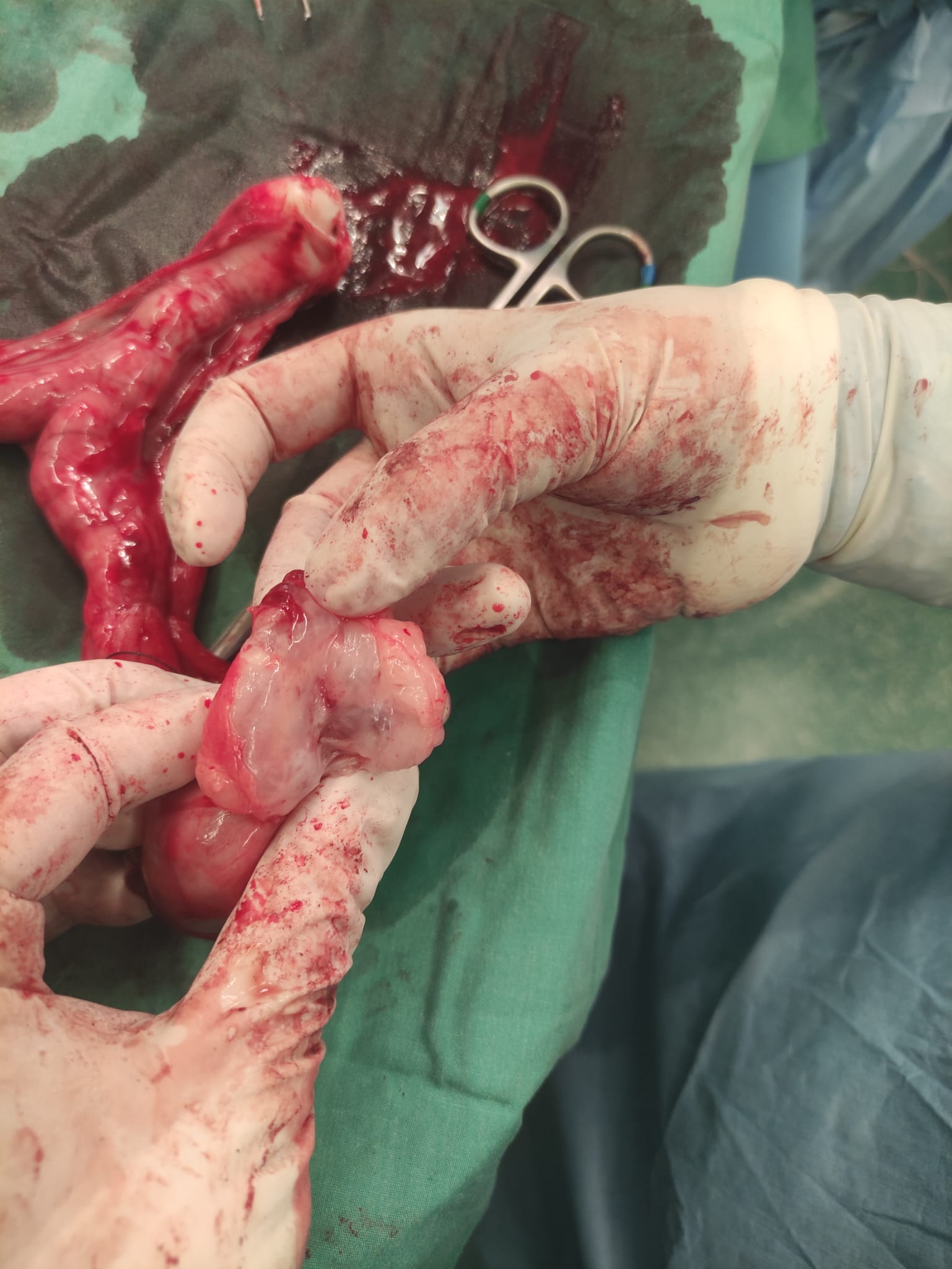

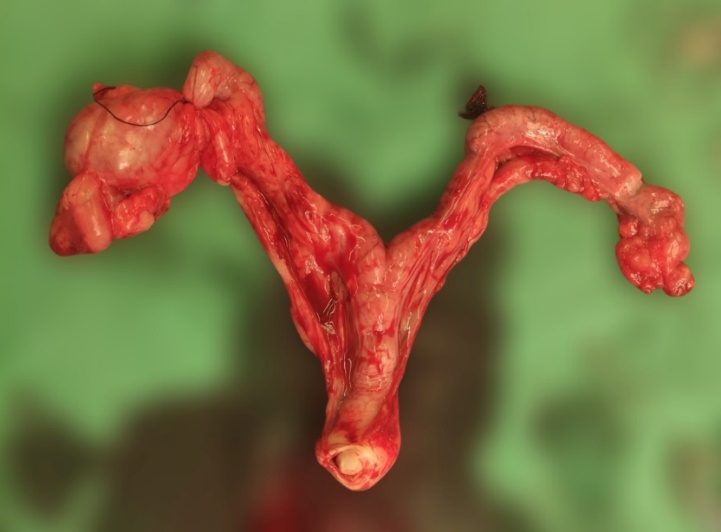


**Supplementary Figure 2.** Macroscopic aspect of the whole reproductive tract after surgical removal. The uterine body and horns showed a hyperplastic appearance (left). On the cut surface of the tissue contiguous with the uterine horns (right, circled in black on the left image), a homogeneous white edematous tissue with multifocal small cystic-like structures was observed, with no evidence of developed or grossly recognizable ovarian tissue. Suture material was used to identify what was presumed to be the proximal end of the left uterine horn.


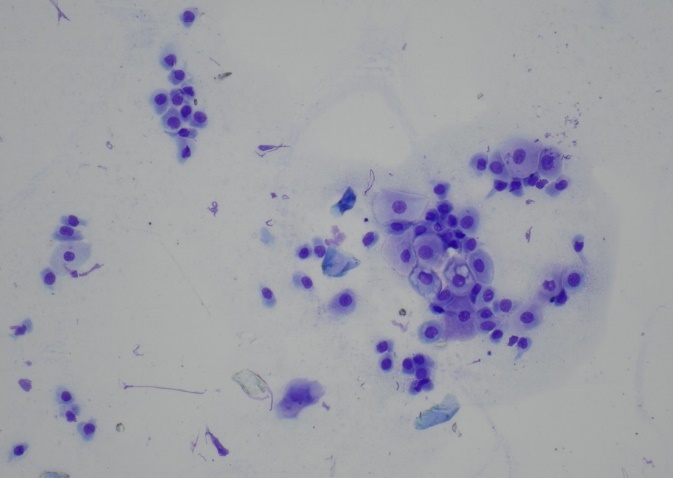


**Supplementary Figure 3.** Vaginal cytology after surgery (100x magnification) compatible with a sterilized bitch with evidence of moderate cellularity, over 90% of non-keratinized epithelial cells (parabasal (black arrow) and intermediate ones (red arrow) to a lesser extent). Erythrocytes and polymorphonucleated cells were not present
